# Nutrition Alters the Stiffness of Adipose Tissue and Cell Signaling

**DOI:** 10.3390/ijms232315237

**Published:** 2022-12-03

**Authors:** Alex Naftaly, Nadav Kislev, Roza Izgilov, Raizel Adler, Michal Silber, Ruth Shalgi, Dafna Benayahu

**Affiliations:** Department of Cell and Developmental Biology, Sackler School of Medicine, Tel Aviv University, Tel Aviv 6997801, Israel

**Keywords:** adipose tissue, high-fat diet, niche stiffness, AGE–RAGE

## Abstract

Adipose tissue is a complex organ composed of various cell types and an extracellular matrix (ECM). The visceral adipose tissue (VAT) is dynamically altered in response to nutritional regimens that lead to local cues affecting the cells and ECM. The adipocytes are in conjunction with the surrounding ECM that maintains the tissue’s niche, provides a scaffold for cells and modulates their signaling. In this study, we provide a better understanding of the crosstalk between nutritional regimens and the ECM’s stiffness. Histological analyses showed that the adipocytes in mice fed a high-fat diet (HFD) were increased in size, while the ECM was also altered with changes in mass and composition. HFD-fed mice exhibited a decrease in elastin and an increase in collagenous proteins. Rheometer measurements revealed a stiffer ECM in whole tissue (nECM) and decellularized (deECM) in HFD-fed animals. These alterations in the ECM regulate cellular activity and influence their metabolic function. HFD-fed mice expressed high levels of the receptor for advanced-glycation-end-products (RAGE), indicating that AGEs might play a role in these processes. The cells also exhibited an increase in phosphoserine^332^ of IRS-1, a decrease in the GLUT4 transporter levels at the cells’ membrane, and a consequent reduction in insulin sensitivity. These results show how alterations in the stiffness of ECM proteins can affect the mechanical cues transferred to adipocytes and, thereby, influence the adipocytes’ functionality, leading to metabolic disorders.

## 1. Introduction

The incentive to study adipose tissue’s function is linked to its essential role in metabolism and its involvement in lipid metabolic complications and disorders [1,2]. White adipose tissue (WAT), the predominant type of fat in the body, profoundly affects metabolism and control of energy homeostasis. The WAT is subdivided according to its anatomical location into subcutaneous fat (SAT) and visceral adipose tissue (VAT) [3,4]. In this study, we focus on the VAT, which is responsible for regulating energy dynamics. It is also closely associated with insulin signaling and the development of insulin resistance (IR) which occurs as part of the metabolic syndrome [5,6].

The complex assortment of cellular populations comprising the adipose tissue mesenchymal populations, including adipocytes, mesenchymal stem and progenitor cells, together with endothelial and immune cells, such as macrophages and lymphocytes. These cell populations constantly interact to regulate the tissue’s activity [3,7]. 

In addition to cells, tissues are composed of an extracellular matrix (ECM), a major niche component with great importance to the microenvironment. In VAT, its composition profoundly influences adipocyte fate and metabolism [8,9] as it generates the 3D architecture of the adipose tissue [10] and serves as a scaffold for the cells in the niche. Alterations in the ECM protein’s profile and structural changes can be facilitated by forming advanced glycation products (AGEs) from protein cross-linking via a non-enzymatic glycation process [11]. As we recently reported for serum albumin, the formation of AGEs and subsequent protein cross-linking increases the stiffness of the protein [12]. In cellular models and tissues, the accumulation of AGEs affects their activity, signaling pathways, and function [13]. Adipocytes, the most abundant cells in fat depots, specialize in their ability to store energy as triglycerides. They actively decrease glucose levels in the circulation via crosstalk between insulin, glucose transporters (GLUTs), and lipid droplets that store it as triglycerides [4,14]. Alteration in their activity can lead to increasing AGEs formation and consequently to further modifications in the ECM niche structure that affect not only the adipocyte’s function but also the whole body’s physiology [11,15].

Adipocytes respond to biochemical and mechano-signals from their environment, which are translated into altered cell signaling [16,17,18,19]. Both these stimuli are affected by the ECM and specifically by the stiffness of the extracellular environment [20,21,22]. We hypothesized that a high-fat diet (HFD) might affect not only the nature and composition of the ECM in adipose tissue but also alter the stiffness of the tissue. The tissue’s modification and remodeling could then affect cell signaling. To investigate this possibility, we used rheology to measure the tissue’s stiffness following HFD nutrition. In addition, we examined the insulin receptor substrate (IRS) phosphorylation levels since an increase in pIRS is related to insulin insensitivity that can lead to IR [23]. From our results, we can conclude that changes in ECM composition and stiffness in response to a HFD affect cell metabolism and may be the leading cause of IR and diabetes pathophysiology. 

## 2. Results

**Figure 1:**
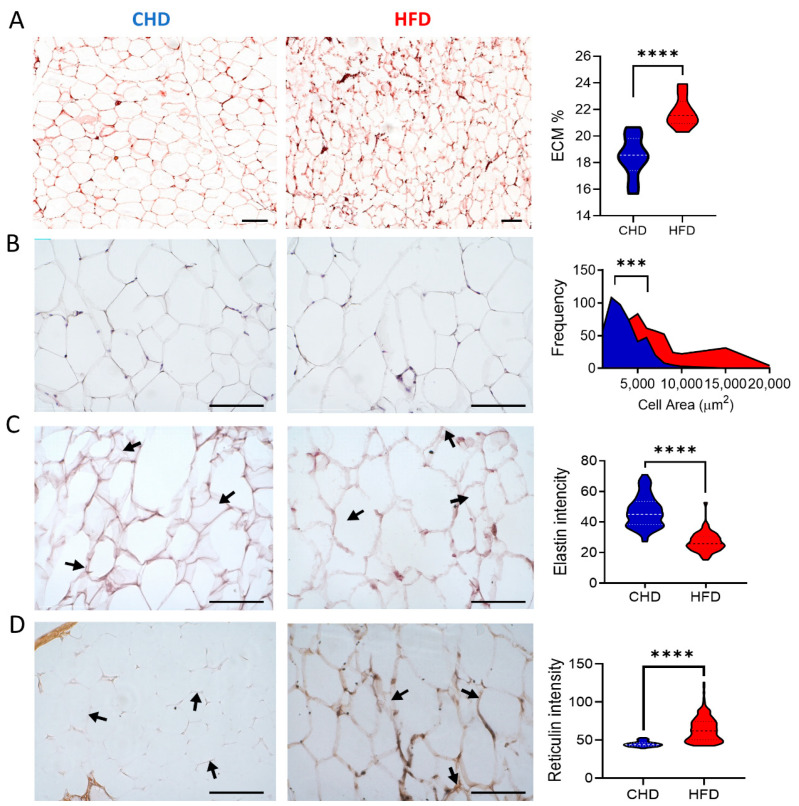
Histology analysis of CHD and HFD-fed mice. (**A**) VAT stained with Hematoxylin and Eosin (H&E) determines ECM percentage (**B**) Adipocytes’ cell size and distribution were quantified for the CHD and HFD-fed mice. (**C**) Orcein stain for elastin fibers in the ECM, expression for CHD and HFD-fed mice. (**D**) Silver stain for reticular fibers in the ECM, intensity is quantified in CHD and HFD-fed mice. For all analyses N = 7 and data presented as means; statistical significance was analyzed by a two-tailed, unpaired Student’s *t*-test, (*** *p* < 0.001, **** *p* < 0.0001), and the scale bar = 100 µm.

**Figure 2:**
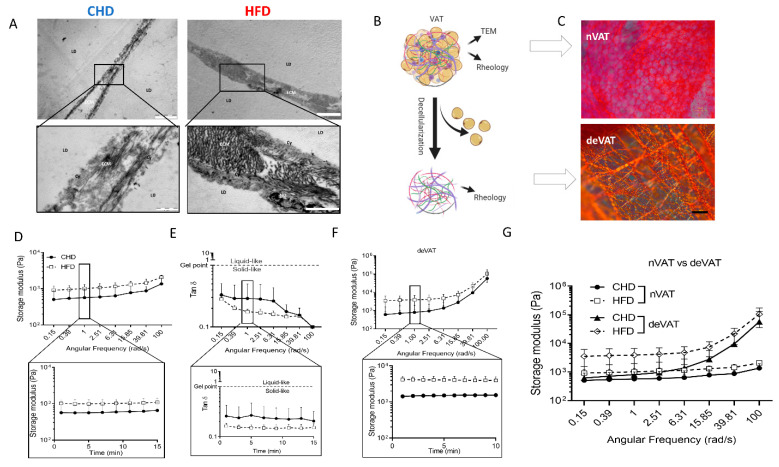
ECM mass and stiffness increase in VAT from HFD-fed mice. (**A**) TEM images of VAT to visualization of LDs, a thin layer of cytoplasm (Cy), and the extracellular space. Lower pictures show the ECM at high-density fibrils from HFD-fed mice, (Scale bar is 5 µm for top images, 1 µm for CHD, and 2 µm for HFD-fed mice at the bottom.) (**B**) Schematic illustration of nVAT and deVAT followed collagen staining and measuring of viscoelasticity and stiffness. (**C**) Picrosirius Red Staining of nECM and deECM collagen fibers in VAT (scale bar = 100 µm) (**D**) Storage modulus represented by logarithmic range dependent on the angular frequency sweep of mice under HFD (empty symbol, n = 5) and CHD (solid, n = 4) and the storage modulus over time under a constant angular frequency of 1 rad/ (**E**) Tan δ is dependent on angular frequency sweep or measured under angular frequency of 1 rad/s over time. The dashed grey line represents the gel point in Tan δ. (**F**) The storage modulus of decellularized VAT is dependent on angular frequency sweep for HFD (n = 7) and CHD (n = 6) and the storage modulus overtime under a constant angular frequency of 1 rad/s. (**G**) The storage modulus of nVAT compared to deVAT represents ECM stiffness. Empty symbols represent VAT from HFD-fed mice, while solid symbols are from CHD-fed mice. The error bars are the standard deviation, and the statistical significance is * *p* < 0.05. The *y*-axis represents the logarithmic axis in the rheology properties results.

**Figure 3:**
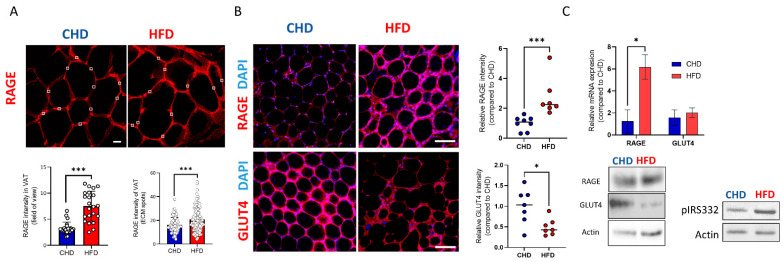
Expression of RAGE, GLUT4, and IRS-1. (**A**) Immunofluorescence staining of RAGE (red) in visceral adipose tissue (VAT) of histological sections from HFD and CHD-fed mice (magnification of ×630, scale bar = 50 μm) and intensity quantification of RAGE expression per field of view or by random points of ECM. (**B**) Whole mount staining and FOV quantification of expression intensity, for RAGE (upper panel) and GLUT4 (lower panel) for HFD (red) and CHD-fed mice (blue), VATs (magnification of ×200, scale bar = 115 μm), Cell nuclei are marked by DAPI (blue). (**C**) Quantitative PCR of VAT analyzed from CHD (blue) and HFD-fed mice (red) (n = 4 per group) for RAGE and for GLUT4 (* *p* < 0.05, *** *p* < 0.001), significance was calculated by an unpaired Student’s *t*-test. Western blot analysis of RAGE, GLUT4 and pIRS-1^Ser332^ in CHD and HFD-fed mice.

We have previously reported that HFD-fed mice exhibit impaired glucose tolerance with increased glucose levels on glucose tolerance tests [24]. In the current study, we focused on the effects of HFD on VAT-ECM to investigate whether the distribution, structure, and composition of ECM with altered stiffness affect the tissue’s function. We used VAT sections from CHD and HFD-fed mice for histological staining and transmission electron microscopy (TEM) to detect changes in ECM proteins (Figure 1 and Figure 2). Rheological measurements of tissue pieces were used to examine their ability to resist deformation and define their stiffness (Figure 2). To inspect the relationship between the altered stiffness, tissue function and AGEs formation, we used immunofluorescence (IF), qPCR, and Western blots (WB) to monitor the expression of signaling receptors (Figure 3).

Histological analysis of paraffin-embedded VAT sections enables visualization of the tissue and ECM by light microscopy (Figure 1). This method was used to detect ECM alterations following the changes in nutritional status and to monitor the remodeling and physiological status of the tissue. Hematoxylin and eosin staining revealed an increase in ECM mass in HFD-fed mice, with a value of 23% of the field of view (FOV) compared to 18% in the CHD-control animals (Figure 1A). At a higher magnification, it is possible to examine the morphology and size of the adipocytes. The results of this analysis indicated that the cells were larger in HFD-fed animals, with a mean of 3062 ± 189.5 µm^2^ for CHD mice and 5768 ± 428 µm^2^ for HFD-fed animals (*p* < 0.0001, Figure 1B). Single-cell analysis revealed a differential distribution pattern in adipocyte size, ranging from 3000 to 20,000 µm^2^ for HFD-fed mice and up to 8000 µm^2^ for CHD-fed animals (measured 460 cells in n = 7, Figure 1B). Other histological staining outlined the ECM components by orcein stain that detect elastin (Figure 1C) and silver stain for reticular fibers that highlight collagen type III. Orcein staining for elastin revealed a 1.75-fold higher intensity in CHD-fed mice (46.9 ± 1.2 measured at n = 76 points) as compared to HFD-fed mice (26.9 ± 0.8, measured at n = 56 points, *p* < 0.0001, Figure 1C). The intensity of reticular fibers was 1.42-fold higher in VAT sections from HFD-fed mice (63.9 ± 1.1 measured at n = 106 points) than in CHD-fed controls (44.9 ± 0.3, measured at n = 200 points, *p* < 0.0001, Figure 1D) in silver-stained tissues. These results indicate that nutrition alters the composition of the ECM. 

The changes in ECM organization were further analyzed by TEM (Figure 2A) and rheology assays were performed to examine the effects on tissue stiffness (Figure 2D–G). TEM micrographs of VAT allow us to visualize the ECM organization in the interstitial space between the cells at high resolution. As shown in Figure 2A, collagen fiber bundles were present at a higher quantity and greater densities in HFD-fed compared to the CHD-fed mice. 

Since the ECM composition was altered, we analyzed the tissue stiffness using rheology measurements under increased or constant angular frequency conditions. Our goal, using the different measurements, was to quantify the tissue stiffness and viscoelasticity of both the native VAT (nVAT) and decellularized VAT (deVAT). Schematic illustration Figure 2B and Picrosirius Red stained collagen fibers visualized in nECM and deECM, Figure 2C. The viscoelasticity analysis revealed that the storage modulus in nVAT from HFD-fed mice at 1 rad/s for 15 min was almost 2-fold higher in HFD than in CHD-fed mice, and remained stable throughout the measurement period (Figure 2D). The gelation point (which reflects a solid or liquid-like behavior) measured by the loss/storage modulus ratio and represented by Tan δ (phase shift) was similar for all animals (Tan δ < 1). The nVAT from HFD-fed mice was more solid than in the CHD-controls, especially at an angular frequency of 0.15 to 15.85 rad/s (Figure 2E). Decellularized ECM (deECM) stiffness measurements revealed a 2.66-fold increase in the storage modulus in HFD-fed mice than in CHD-fed mice at a frequency of 0.15 rad/s that remained constant over time. However, increasing the angular frequency to 15.85 rad/s reduced this difference to only a 1.5-fold higher intensity in the HFD-fed mice (Figure 2F). Interestingly, when comparing nVAT vs. deVAT, the storage modulus shows that the deVAT is more than 5-fold stiffer than nVAT (Figure 2G). The rheological properties reflect a remarkable modification in the ECM in HFD-fed mice, which causes tissue stiffening. 

Next, we sought to examine whether AGEs formed in the adipose tissue in response to HFD nutrition are linked to the VAT stiffening phenomenon. We measured the levels of RAGE, which is a receptor for AGEs (Figure 3) and whose presence indicates an increased level of AGEs. Immunostaining of whole mount (WM)-VAT or paraffin-embedded tissue sections revealed a higher level of RAGE in HFD-fed mice (Figure 3A,B). The levels of RAGE expressed were several folds higher on isolated VAT from HFD-fed mice stained by IF of WM (Figure 3B). Quantification of the RAGE intensity per FOV in the histological sections (Figure 3A) demonstrated a 2.5-fold increase in the HFD-fed animals (7.51 ± 2.85, n = 22 points) compared to CHD-fed mice (3.31 ± 1.15, n = 23 points, *p* < 0.0001). In addition, the intensity measured at random points in VAT histology sections was higher for HFD-fed animals (20.73 ± 9.98, n = 89) than in the control group (6.05 ± 6.36, n = 111 points, *p* = 0.0002). The increased RAGE expression in HFD-fed mice indicates the high level of AGEs present in the VAT of HFD-fed mice. An opposite pattern was observed in whole mount staining of GLUT4 with a 50% reduction in its levels in HFD-fed mice (Figure 3B). The IF results were confirmed by qPCR and WB, where RAGE was shown to be upregulated in HFD-fed mice while GLUT4 was down-regulated (Figure 3C). Additionally, the results revealed higher levels of pIRS-1^Ser332^ in VAT from HFD-fed mice than in the CHD-controls. This, together with the reduced levels of GLUT4, indicated that the adipose tissue from HFD-fed mice is less insulin sensitive, as IR is associated with elevated levels of pIRS-1^Ser332^ [25]. These results indicated that the increase in tissue stiffness might be due to the presence of AGEs in the VAT. The AGEs products, caused by the nutritional regimen, can possibly affect the insulin-signaling pathway. 

## 3. Discussion

Nutritional stimuli affect the adipose tissue mass and may lead to obesity. This, in turn, affects systemic metabolic homeostasis and causes adipose dysfunction, leading to insulin resistance, dyslipidemia, and hypertension, among other problems, which together now represent a major public health pandemic [26,27]. The adipocytes in the tissue play a role in energy storage and are located in a niche composed of various ECM proteins. The increase in WAT mass seen in obesity is a consequence of the increase in the size of the uni-lobular lipid droplets in adipocytes that comes in response to metabolic activity. Both cell growth of existing adipocytes, as well as hyperplasia, which is the formation of new adipocytes through differentiation of pre-adipocytes, contribute to tissue expansion [28]. Cells in the HFD-fed mice exhibited hypertrophy, a process of increment in the volume of existing cells (Figure 1) also reported in other studies [29], a phenomenon that was observed in obese humans [30,31,32]. 

Adipocytes are embedded in an ECM scaffold, which provides the signals regulating the tissue’s architecture and functionality of the cells; thus, studying the changes in the ECM composition is of prime importance. We have previously reported on ECM-related changes that occur during adipogenesis in vitro [8]. Here, we examined the cellular and ECM components of adipose tissue in response to the nutritional of HFD feeding in mice. When prominent changes occur, the ECM affects the signaling of adipose cells, contributing to the vicious cycle and interruptions in insulin signaling [33]. Histological analysis of the adipocyte’s niche revealed changes in ECM components, with a reduction in elastin and an increase in collagenous proteins in HFD-fed mice (Figure 1). High-resolution TEM imaging of VAT from HFD-fed mice demonstrated an increase in the mass and density of ECM components compared to CHD-fed mice (Figure 2). The changes in the arrangement of collagen fibers in the ECM were seen in macro histology, where the fibers were shown to form a mesh around the cells (Figure 2C), as well as in microscale TEM (Figure 2A). It was previously demonstrated that various collagens, such as collagen type I or VI, were upregulated in VAT of HFD-fed mice and db/db mice [34,35,36,37]. Collagen type III is also increased in HFD-fed mice and upregulated in db/db mice [38], and an increase in collagen XVIII was noted in human obese [39,40]. 

In contrast, a reduction in the elastin in the adipose tissue has been associated with glucose uptake interference, and the induction of IR and type 2 diabetes [41,42]. The excessive deposition of ECM components in adipose tissue of HFD-fed mice (Figure 1 and Figure 2), along with the changes in the proteins deposited, affected the ECM properties and led us to examine the tissue stiffness and viscoelastic properties (Figure 2). The convergent approaches in this study present a novel concept concerning the changes in tissue stiffness due to protein content modifications and an elevated AGEs formation in HFD-fed mice. The observed altered ECM, caused by nutritional effect, led to alterations in VAT’s stiffness when comparing the HFD to CHD-fed mice, which in turn had a profound impact on the mechano-biological properties of the niche. The notion that changes in protein composition can affect the ECM mechanical properties was suggested in our earlier studies. We have shown in silico and by physical measurements that adipocyte and niche stiffness increase with lipid droplets’ accumulation [43,44,45,46,47]. In this study, we focused on the ECM of fatty tissues. Adipocytes in HFD-fed mice have larger lipid droplets, and larger cells influence the mechanical properties of the tissue. Therefore, to isolate the effect of the ECM, we separated the deECM, the cells’ niche from the whole tissue (nECM) (Figure 2B,C) and tested the stiffness without the impact of adipocytes. In the rheology analysis of nECM and deECM (Figure 2D–G), the HFD-ECM was stiffer than ECM retrieved from CHD-fed mice. This phenomenon was more prominent in the deECM with its solid-like behavior (Figure 2G). The nECM and deECM were shown to contain various proteins with some notable collagen constituents that have a major role in the ECM-formed fibers [3,8,19]. These fibers undergo crosslinking with aldehyde and ketone groups’ derivatives of sugar and form AGEs. Additional ECM components can also crosslink into AGEs, such as laminin, fibronectin, and elastin [48,49]. Nevertheless, collagen is a significant glycation target in the ECM and is known to affect the functionality and mechanical properties of the tissues [11,50,51]. 

The viscoelastic and stiffness properties analyses revealed an increase in tissue stiffness resulting from an HFD nutrition intertwined with the formation of AGEs. HFD consumption leads to increased AGEs formed and the production of more ECM mass than seen in CHD-fed mice. Interestingly, an increase in AGEs formation in VAT has been observed in obese diabetic patients [11], and such alterations in tissue properties have also been associated with cardiovascular complications that were attributed to increased cell niche stiffness [52,53,54]. 

With this important finding of changes in the tissue’s mechanical properties, the accumulation of AGEs in HFD-fed mice is mediated by increased expression of RAGE detected by immunofluorescence, qPCR, and WB. The accumulation of AGEs triggers the AGE–RAGE axis and induces an elevation in the RAGE expression, a core driver of the AGEs signaling pathway. The elevation of the AGE–RAGE axis in response to HFD nutrition has also been reported in granulosa cells (GCs) [24] and a variety of other cells in vitro [55,56,57]. Here, we suggest that elevation in RAGE expression is associated with the formation of AGEs [58,59], and a stiffening of the cell niche may represent a novel mechanism for insulin resistance. Evidence for this is derived from the increase in pIRS-1^Ser332^ in the VAT from HFD-fed mice and the concomitant decrease in GLUT4 activity demonstrated by immunofluorescence and WB (Figure 3). The role of pIRS-1 in the development of IR is well accepted in ob/ob mice and the HFD-induced obesity model [25,60]. 

In summary—Feeding mice with HFD causes the adipose cell niche to become stiffer due to alteration in ECM proteins and the formation of AGEs. This, in turn, leads to an increase in RAGE and pIRS-1^Ser332^ expression levels and a concomitant decrease in GLUT4 expression levels. This induces a reduction in insulin sensitivity in HFD-fed mice. We can conclude that HFD nutrition leads to the formation of AGEs, and increased tissue stiffness that mediates the increased risk of obesity and IR in adipose tissue. 

Perspective—In this study, we provide a better understanding of the crosstalk between nutritional effects and ECM changes where increased stiffness alters adipocyte signaling resulting in elevated pIRS and a concomitant decrease in GLUT4 expression, leading to the development of IR.

## 4. Materials and Methods

### 4.1. Animals

ICR female mice (Envigo RMS Limited, Jerusalem, Israel) were housed in temperature- and humidity-controlled rooms at the animal facility of Tel Aviv University (TAU), under artificial illumination for 12 h daily; food and water were provided ad libitum. They were fed (6–14 weeks old), with either a regular diet or HFD (60% calories from fat; Teklad 06414, Envigo RMS Limited). Upon euthanasia of the mice at 14 weeks of age, visceral adipose tissue (VAT) was collected and used either as fresh, or frozen by liquid nitrogen)kept at −80 until use) or fixed for further procedures. Animal care and all experiments were by the guidelines of the IACUC, TAU Approval (01-15-093) [24].

### 4.2. Histology and Immunofluorecence Staining and Analysis

Histology staining: VAT was immediately fixed in 4% paraformaldehyde overnight at 4 °C, washed with PBS, ethanol, and xylene, then embedded into paraffin blocks to prepare five µm thick sections. For staining, sections were deparaffinized, rehydrated, and stained with Hematoxylin-Eosin, Orcein and Silver stains and visualized by light microscopy (Nikon Optiphot-2, Nikon, Tokyo, Japan). Single cell’s area and the ECM fraction in the tissue were analyzed from various FOVs. Images were converted into a binary format to calculate the percentage of ECM in each FOV.

Immunofluorescence staining: Paraffin-embedded sections were stained for RAGE (SC 365154) (Santa Cruz Biotechnology, Dallas, TX, USA) and with secondary antibody Cy3 Goat anti-Mouse (Jackson ImmunoResearch Laboratories, West Grove, PA, USA; 115-165-003). Images of the stained sections were acquired by a fluorescence microscope (Eclipse Ci; Nikon, Tokyo, Japan) (×400). All images were processed and analyzed using the ImageJ software (NIH, Bethesda, MD, USA). 

### 4.3. Picrosirius Red Staining for Collagen Fibers

The VAT was stained in Picrosirius Red solution (0.1% sirius red 0.1% in saturated picric acid) for 1 h, then rinsed in acidic water until clear, and dehydrated in ethanol 3 times. This staining method combined with polarization enables imaging of birefringent collagen fibers [3,61] (Nikon Optiphot-2, Tokyo, Japan).

### 4.4. Transmission Electron Microscopy (TEM)

VAT was fixed overnight in 2.5% Glutaraldehyde in phosphate-buffered (PBS) at 4 °C was then washed several times with PBS and post-fixed in 1% OsO4 in PBS for 2 h at 4 °C. Dehydration was carried out in graded ethanol and embedded for preparation of thin sections mounted on Formvar/Carbon coated grids. Sections were stained with uranyl acetate and lead citrate and examined using a JEM 1400 Plus transmission electron microscope (Jeol, Akishima, Tokyo, Japan). Images were captured using SIS Mega view III and the TEM imaging platform (Olympus, Shinjuku City, Tokyo, Japan) [3]. 

### 4.5. Tissue Extracellular Matrix (ECM) and Decellularization

VAT was decellularized (deVAT) according to the Wang protocol [3,62]. Briefly, three freeze and thaw cycles were performed, followed by an extensive wash in double-distilled water (DDW) for 2 days at RT with agitation at 120 rpm. The tissue was washed in 0.5 M NaCl and 1 M NaCl for 4 h each and then with DDW overnight. Next, tissue was digested with 0.25% trypsin-EDTA washed, and incubated with isopropanol overnight. Afterward, samples were treated with 1% Triton X-100 for 3 days (changed daily), rinsed with PBS and stored in isopropanol. 

### 4.6. Rheological Measurements

Dissected VAT from CHD and HFD-fed mice were measured as was previously described [12]. The tissues were measured on an HR-3 hybrid Rheometer (TA Instruments, New Castle, DE, USA) used a 8-mm diameter parallel Peltier plate at 25 °C or 37 °C according to ISO 6721-1 method to measure the viscoelasticity and determine the rheological properties of VAT. The tissues were measured for storage (G′) and loss modulus (G″) and monitored for Tan δ as a function of angular frequency either between 0.1–100 rad/s and a strain between 0.8–1 percent. An overtime test with a constant angular frequency of 1 rad/s at 37 °C was used to observe the transformation from liquid to solid-like behavior.

### 4.7. Whole-Mount immunofluorescence staining

VAT whole-mount staining was performed as described [3]. The VAT samples were stained with primary antibodies; Glucose transporter 4 (GLUT4; SC 53566), and RAGE (SC 365154), both from Santa Cruz (Santa Cruz Biotechnology, Dallas, TX, USA). Next, the tissues were incubated with secondary antibodies; Cy3 Goat anti-Mouse (Jackson ImmunoResearch Laboratories, West Grove, PA, USA; 115-165-003) and Fluor 555 anti-Mouse IgG1 (Invitrogen, Waltham, MA, USA; A-21127). Before visualization, a fluoroshield mounting medium containing 4′,6-diamidino-2-phenylindole (DAPI) (Electron Microscopy Sciences, #17985-10) was added to the tissues, which were then viewed and imaged by a confocal SP8 microscope (Leica SP8; Leica, Wetzlar, Germany).

### 4.8. Biochemistry and Protein Identification Western Blot (WB)

Protein extraction from VAT and immunoblotting were performed as previously described [63]. Tissues were collected, washed with ice-cold PBS, and lysed in 50 mM Tris pH 7.5, 150 mM NaCl buffer containing 1 mM EDTA, 1% NP-40, and protease inhibitors (200 mg of tissue per sample). Protein concentration was determined with BCA Protein Assay Kit (Pierce Biotechnology, Rockford, IL, USA, 23225). Samples were re-suspended in Laemmli buffer, separated on 7.5% SDS–PAGE gel, transferred to nitrocellulose, and incubated overnight with primary antibodies GLUT4 (SC 53566), RAGE (SC 365154), Actin (SC 47778) (Santa Cruz Biotechnology, Dallas, TX, USA), and rabbit anti-pIRS-1^Ser332^ [25]. They were washed and incubated with Peroxidase Anti-Mouse IgG (115-035-003) and Peroxidase Anti-Rabbit IgG (111-035-003), both from Jackson (Jackson ImmunoResearch Laboratories, West Grove, PA, USA) in blocking solution. Peroxidase signal was detected with chemiluminescent substrate (Pierce Biotechnology, Rockford, IL, USA) using Fusion FX7 (Vilber, Collégien, France). 

### 4.9. RNA Isolation and qPCR 

Total RNA was extracted from VAT (200 mg of tissue per sample) (BioTri, Bio-lab Ltd., Jerusalem, Israel) and reverse transcribed to cDNA using High-Capacity cDNA Reverse Transcription Kit (Applied Biosystem, Waltham, MA, USA). Transcripts levels were measured with SYBR green (Applied Biosystem, Waltham, MA, USA) using STEPONE plus system (Thermo Fisher Scientific, Waltham, MA, USA). All data were normalized to actin by the delta-delta Ct method. 

The sequences of the primers are:
**Gene****Forward Primer****Reverse Primer**ActinCATCGTGGGCCGCCCTAGGCACCACGGTTGGCCTTAGGGTTCAGGGGGRageAACACAGCCCCCATCCAAGCTCA CCAACAGCTGAATGGlut4TTCACGTTGGTCTCGGTGCTTAGCTCATGGCTGGAACCCG

### 4.10. Statistical Analysis

Statistical analysis was performed using a two-tailed, unpaired, *t*-test to identify significant differences in the samples. For each parameter, it was presented as the means and standard error, and *p* < 0.05 was considered statistically significant. Distribution charts and plots were performed using Graph Pad Prism 9 software.

### 4.11. Schematic Illustrations

Schematic Illustrations were created by the Bio Render software https://biorender.com (accessed on 18 September 2022).

## Data Availability

Data are available from the authors upon reasonable request.
